# Species diversity within morpho-functional groups drives diversity dependence in reef ecosystems

**DOI:** 10.1371/journal.pone.0338441

**Published:** 2025-12-17

**Authors:** Anupama Chandroth, Claudia C. Johnson

**Affiliations:** Department of Earth and Atmospheric Sciences, Indiana University Bloomington, Indiana, United States of America; National Cheng Kung University, TAIWAN

## Abstract

Changes in species diversity can alter reef ecosystem composition by impacting the origination and extinction of species within co-existing groups. A massive literature exists on exploring the impact of species diversity shifts through a taxonomic centered perspective. We here address the consequences of shifts in diversity using a novel morpho-functional (MF) group approach. We categorized species into MF groups based on growth forms. Our analysis demonstrates that species diversity has not been uniform across morpho-functional (MF) groups over time. Examination of coral origination and extinction rates revealed multiple pulses of rate shifts, indicating non-uniform rate changes in both processes. When addressing the potential drivers for these patterns, we found no significant correlation between species diversity increases and extinction within MF groups, thus identifying the absence of redundancy-related effects within individual groups. However, our study highlights a strong correlation between species diversity within a MF group and the rates of origination and extinction of some co-existing groups, thus, identifying morphology mediated species diversity as a potential driver for diversity dependence. We demonstrate this relationship is selective and that species capable of building biogenic habitat structures can influence the diversity and morphology of species that coexist, thus constantly modifying the reef architecture and functional potential through time. Species diversity dependence mediated through morphology shapes the reef functional composition through time.

## Introduction

Changes in species diversity can trigger a chain reaction often mediated by competitive or facilitative interactions [1] that lead to decline of species or to origination of new species [2,3]. Species diversity dependent impacts have been explored across clades [4], clades and taxonomic groups [5], guilds [6] and functional groups [7] and it has been established that species diversity in one such group can impact the diversity in other groups. While morphology is recognized as a factor influencing species interactions [8], no study has examined how morphology alone shapes species diversity dependencies and ecosystem functioning, thus leaving a gap in understanding the role of morphology in mediating biodiversity patterns. To identify the impact of species diversity associated with morphology, we focus on the effects of species diversity within morpho-functional (MF) groups on extinction and origination. This MF approach examines the role of clade-independent morphology in shaping macroevolutionary patterns in a given ecosystem. While our results support the research of previous workers in establishing diversity-dependence (e.g., [3,4,9,10], our unique MF group approach also reveals species diversity mediated through morphology as a driver of diversity-dependence.

**Fig 1:**
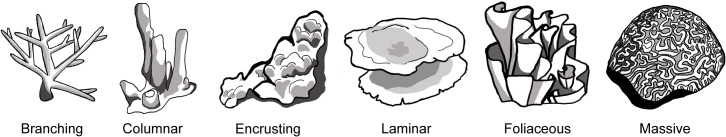
Types of growth forms used in this study. In this study we have used 6 colonial growth-forms as described by Veron (2000) to classify the coral species to morpho-functional (MF) groups (Growth form images are hand drawn by the authors using reference images from the Caribbean reefs taken by the authors).

To identify the role of morphology in shaping species diversity-dependent relationships, we propose a clade independent categorization of species into morpho-functional groups based solely on species morphology. There is conflicting evidence for phylogenetic relatedness capturing functional and morphological similarities [11–13]. Species within the same clade can have different morphologies, contributing differently to ecosystem functioning [14–17]. Especially for ecosystem constructors such as plants, molluscs, and corals, morphology rather than the clade association dictates their function in an ecosystem [18–21]. In a reef ecosystem different coral species within the same group family, genus or even clade can have different growth forms [19,22,23] and it is the coral growth forms that dictate the range of ecosystem functions that a species is capable of performing, including environmental adaptation, primary productivity, resource competition, and habitat construction [17,24–26]. At one end of the spectrum of growth forms, branching corals exhibit high surface complexity, contributing to reef matrix infilling and providing crucial habitat refuge for various reef species; the massive corals, positioned at the opposite end with low surface complexity, can enhance reef matrix stability and cause minimal direct competition on other species (Fig 1) [17]. Though functionally diverse, these distinct growth forms are found within the same family or even genus (e.g., extant Poritidae and Acroporidae). Consequently, closely related species have the potential to exhibit different competitive and facilitative interactions with the co-existing species due to differences in growth forms and this can have large scale macroevolutionary implications specifically in the context of diversity dependence.

Here we use the recently developed PyRate algorithm [27] to analyze species diversity dependence mediated by morphology on co-existing scleractinian coral species. We expect that the species diversity within coral MF groups influences the diversity within co-existing groups and thereby alters the overall reef functional composition. Darwin’s theory of competition among “similar species” [28], implies that an increasing species diversity within a group is expected to intensify intra-group competition and limit the group’s origination rate due to functional redundancy. We examine our research expectations using Paleocene through Pleistocene coral fossil records from the Caribbean reef system [22]. With MF groups created using coral growth forms we address 1) the uniform distribution of species across all morpho-functional groups, (2) the rates of extinction and origination of coral species within MF groups through time, (3) the influence of species diversity within a MF group in promoting extinction of species within that group and (4) the influence of species diversity within a MF group impacting the species diversity of other MF groups through extinctions and originations. Outcomes from this research enable us to determine the effect of species diversity held within MF groups in shaping the macroevolutionary patterns that lead to the present-day shallow-water coral reef biodiversity in the Caribbean reef ecosystem.

## Results

### Diversity patterns through time

Our results show that species diversity is not uniform across all morpho-functional (MF) groups through time. Two hundred fifty species of Paleocene through Pleistocene colonial hermatypic zooxanthellae bearing scleractinian corals (hereafter referenced as corals) were organized into 17 MF groups (Table 1). Overall, within MF groups the species diversity varied from 123 to 1. Eight of the groups are functionally vulnerable with only 3 or fewer species, and the other nine groups are identified as the dominant groups (Table 1).

**Table 1:** Morpho-Functional group summary table.

MF group	Proportion of occurrences	Species diversity
Massive (M)	**0.249**	**123**
Branching (B)	**0.234**	**49**
Massive + Encrusting (ME)	**0.209**	**15**
Laminar (L)	**0.033**	**10**
Massive + Laminar (ML)	**0.023**	**10**
Encrusting (E)	**0.01**	**10**
Branching + Massive (BM)	**0.016**	**8**
Massive + Columnar (MC)	**0.123**	**7**
Laminar + Encrusting (LM)	**0.062**	**5**
Branching + Encrusting (BE)	0.016	3
Columnar + Encrusting (CE)	0.005	3
Laminar + Foliaceous (LF)	0.002	2
Laminar + Foliaceous + Encrusting (LFE)	0.01	1
Massive + Laminar + Encrusting (MLE)	0.01	1
Columnar (C)	0.002	1
Foliaceous (F)	0.001	1
Columnar + Laminar (CL)	0.001	1

Total proportion of Occurrence and Species Diversity within each of the 17 Morpho-Functional Group. The abbreviations in parentheses are used in the further tables. Dominant groups included in the diversity dependence analysis are highlighted in bold.

**Fig 2:**
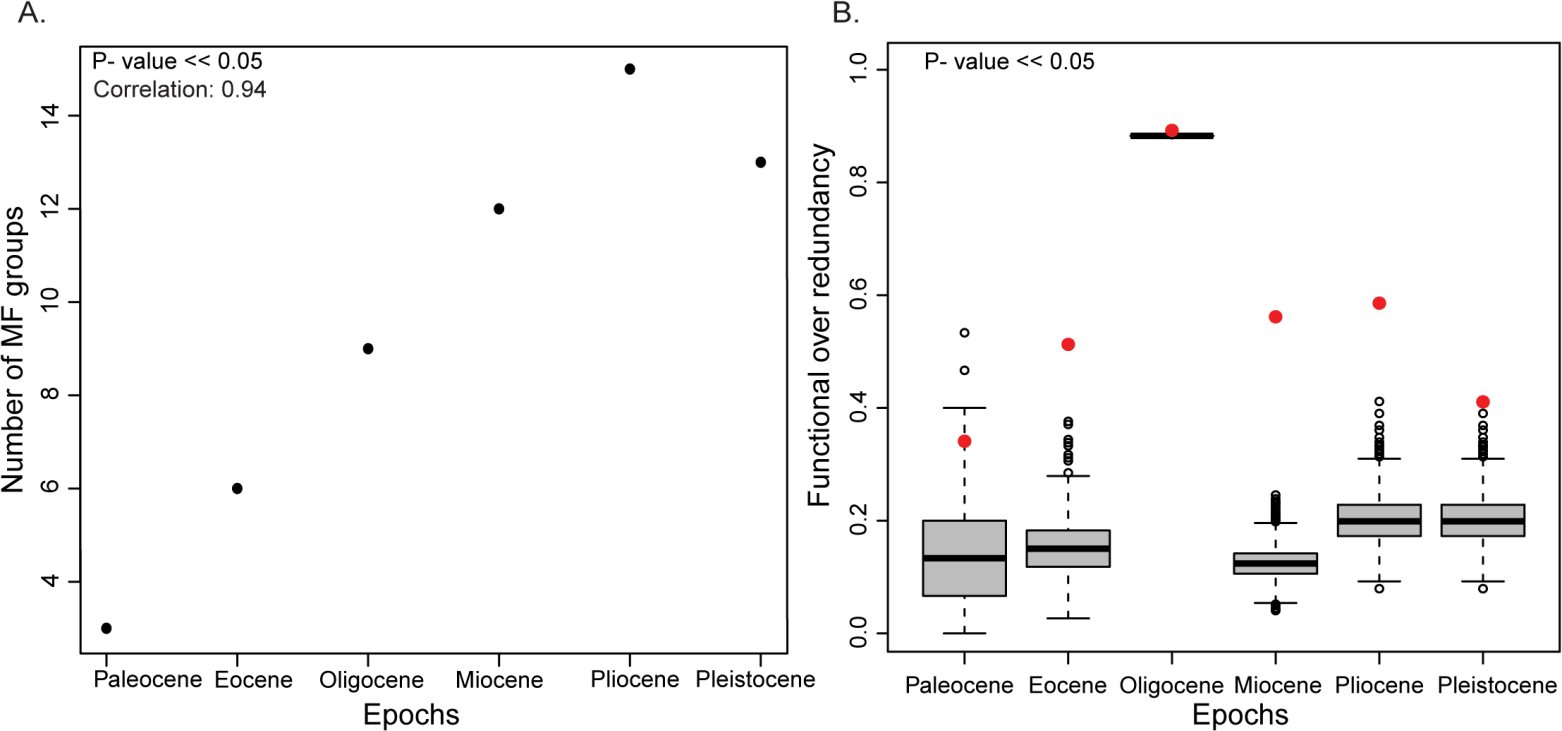
Functional Group Trends Over Time and Comparison of Simulated and Observed FOR Values. The figure shows **(A)** Change in number of functional groups through time and **(B)** Boxplot displaying the range of simulated Functional Over Redundancy (FOR) values, with observed values marked by red circles (p-value>> 0.05).

**Fig 3:**
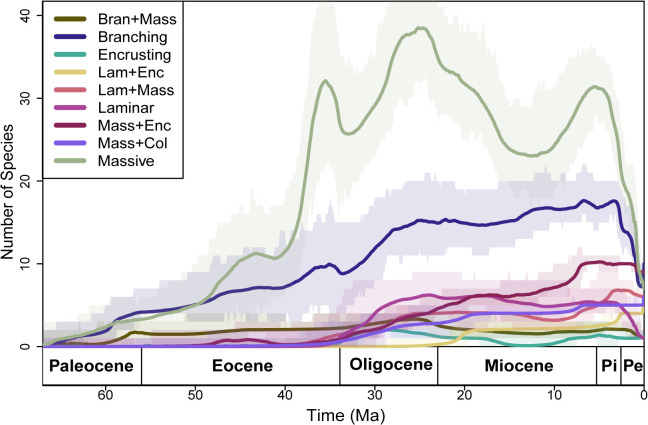
Range-through-time plot for the dominant morpho-functional groups. The range-through-time plot shows the diversity trends for the 9 dominant morpho-functional groups, with diversity calculated at 0.1 Myr increments. The central dark line represents the mean diversity trajectory from 10 replicates, while the shaded area indicates the range of observed values.

The temporal patterns of species diversity and morpho-functional group diversity varied and peaked at different times. We expected a positive correlation between species diversity and the number of MF groups for a uniform distribution of species diversity within MF groups. However, while the number of MF groups showed a significant increase with time (Table 2, Fig 2A), species diversity did not show any positive correlation with time (correlation – 0.48 and p-value – 0.33)(Table 2) and an increase species diversity is not translated to a corresponding change in MF group diversity (correlation- 0.69, p-value- 0.12). The diversity of species declined to nearly half from the Pliocene to Pleistocene, but this resulted in the loss of only two MF groups (Table 2). We used functional indices such as functional redundancy and functional over redundancy to test for uniformity of species packing within MF Groups (Table 2). We observe that at least one third of the species shows overpacking, and the high values of FOR represent a non-uniform distribution of species across all the MF groups within each epoch. The highest levels of FR and FOR were observed during the Oligocene, when the number of unique MF groups remained low despite high species diversity (Table 2, Fig 2B) and the post Oligocene decline in FR and FOR indicates a rise in number of MF groups. The expected random over-redundancy values from the null models were found to be significantly lower than the observed FOR values (p-value< 0.05), resulting in a non-uniform and nonrandom packing of species within each MF group through time (Fig 3).

**Table 2:** Morpho-functional group summary and functional indices.

Epoch	No. species	MF groups *	FR**	FOR ***	Null model Mean+ SD
Pleistocene	47	13	3.65	0.4	0.20 ± 0.04
Pliocene	86	15	5.73	0.58	0.40 ± 0.04
Miocene	111	12	9.25	0.55	0.13 ± 0.03
Oligocene	92	9	10.22	0.88	0.87 ± 0.0002
Eocene	31	6	5.17	0.5	0.15 ± 0.05
Paleocene	15	3	5	0.33	0.14 ± 0.07

Total number of species, morpho-functional groups (MF), functional redundancy (FR), and functional over-redundancy (FOR), along with results from null models based on 9,999 permutations across the 6 epochs (Standard deviation values are included).

### Rate shifts of scleractinian corals

**Fig 4:**
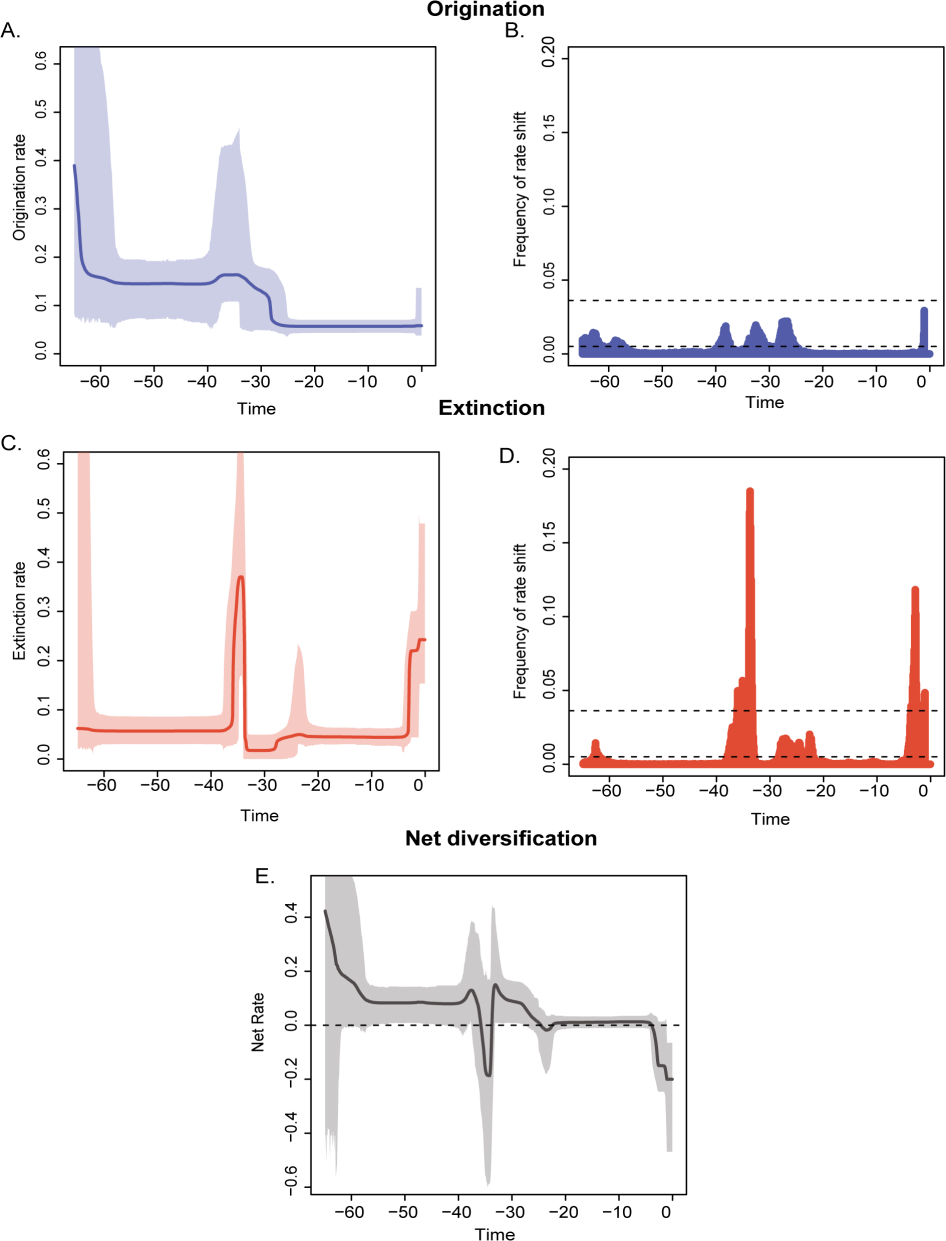
Temporal Dynamics of Origination and Extinction Rates Estimated Using the Bayesian Multivariate Birth–Death Model in PyRate. The figure shows the **(A)** Change in origination rates over time. **(B)** Frequency of origination rate shifts. **(C)** Change in extinction rates over time. **(D)** Frequency of extinction rate shifts. **(E)** Net diversification rates, illustrating the combined effects of origination and extinction dynamics through time. Solid lines indicate mean posterior rates, and shaded areas show 95% CI.

Our exploration of rates of origination and extinction of corals revealed non uniform changes in both origination and extinction rates through multiple pulses of rate shifts. We found three peaks of origination rates and four extinction rate peaks (Fig 4A, 4C). These origination and extinction peaks were first observed in the Early Paleocene at around 65 Mya, followed by a peak between Middle Eocene (Bartonian starting at ~ 39 Mya) to Early Oligocene (Rupelian at ~32 Mya)) and then across the Plio-Pleistocene boundary (Piacenzian to Gelasian (~ 3 Mya to 2 Mya) for origination and Zanclean to Gelasian (~ 5 Mya to 2 Mya) for extinction), and an additional peak during late Oligocene (Chattian at ~ 25 Mya) for extinction alone (Fig 4A, 4C). The peak in origination rate between Middle Eocene (Bartonian at ~ 40 Mya) to Early Oligocene (Rupelian at ~ 24 Mya) is a result of 3 distinct pulses of rate shifts spanning across the same time frame (Fig 4A, 4B). The net diversification remained positive until the Middle Eocene (Bartonian at ~ 38Mya); the second positive peak marked a rise in species diversity along with the accumulation of several new MF groups (Fig 4E, Table 2). However, a drop in net diversification rate declined to a negative scale around the late Eocene (Priabonian at ~35 Mya) and through the Plio-Pleistocene (~ 5 Mya to 1 Mya) due to significant pulses of extinction rate shifts (Fig 4D, 4E). Seven out of the nine dominant groups exhibited rate shifts above the background rate. The exceptions were the Massive+Laminar, Laminar+Encrusting, and Massive+Columnar groups (see S1–S9 Figs). The decline in diversification rate during the Late Eocene mostly coincided with a decline in diversity within the Massive MF group (S1 Fig). No other MF group indicated a high proportion of rate shift during this period (Fig 3, S2–S9 Figs). The Plio-Pleistocene decline of net diversification rate is associated with the decline of Massive, Branching and Laminar MF groups (Fig 3, S1, S2 and S6 Figs). The increased rate shift pulses between ~40 and 30 Ma coincided with a rapid rise in species numbers and the emergence of new functional groups (Table 2, Fig 3). Our study revealed that, beyond the overall diversity trajectory, extinction and origination rates varied across groups, with no two groups following similar patterns.

### Impact of redundancy

There is no significant correlation between the increase in species diversity and extinction within a MF group. We observed that MF groups, such as Massive and Branching, have a high functional redundancy compared to other co-existing groups (Table 1), but this did not lead to a significant promotion of extinction within them. The multi-trait extinction (MTE) analysis examined the impact of growth form categorization and functional redundancy on extinction rates. Both growth-form based MF grouping and redundancy positively influenced extinction rates. However, the relationship was slightly stronger for MF group categorization (log Bayes factor 6) compared to redundancy (log Bayes factor 4) (S10A Fig). However, a further analysis revealed that only two MF groups, Massive and Encrusting, showed a higher-than-expected relative effect on extinction, but with low statistical support (S10B Fig). The ranges of values observed for all the other groups fell within the random expected range for relative impact on extinction, showing the lack of selectivity across those groups. While we expected higher extinction rates with increased redundancy (i.e., increasing species diversity) among coral MF groups, there was no significant difference between categories 1–4 (from the most functionally vulnerable to second highest functionally redundant) (Table 1 and S10C Fig). Only category 5, with exceptionally high redundancy values (32 or more species), showed an elevated extinction rate above the background level (S10C Fig). However, this result had limited statistical support (p-value < 0.05).

### Impact of diversity

**Fig 5:**
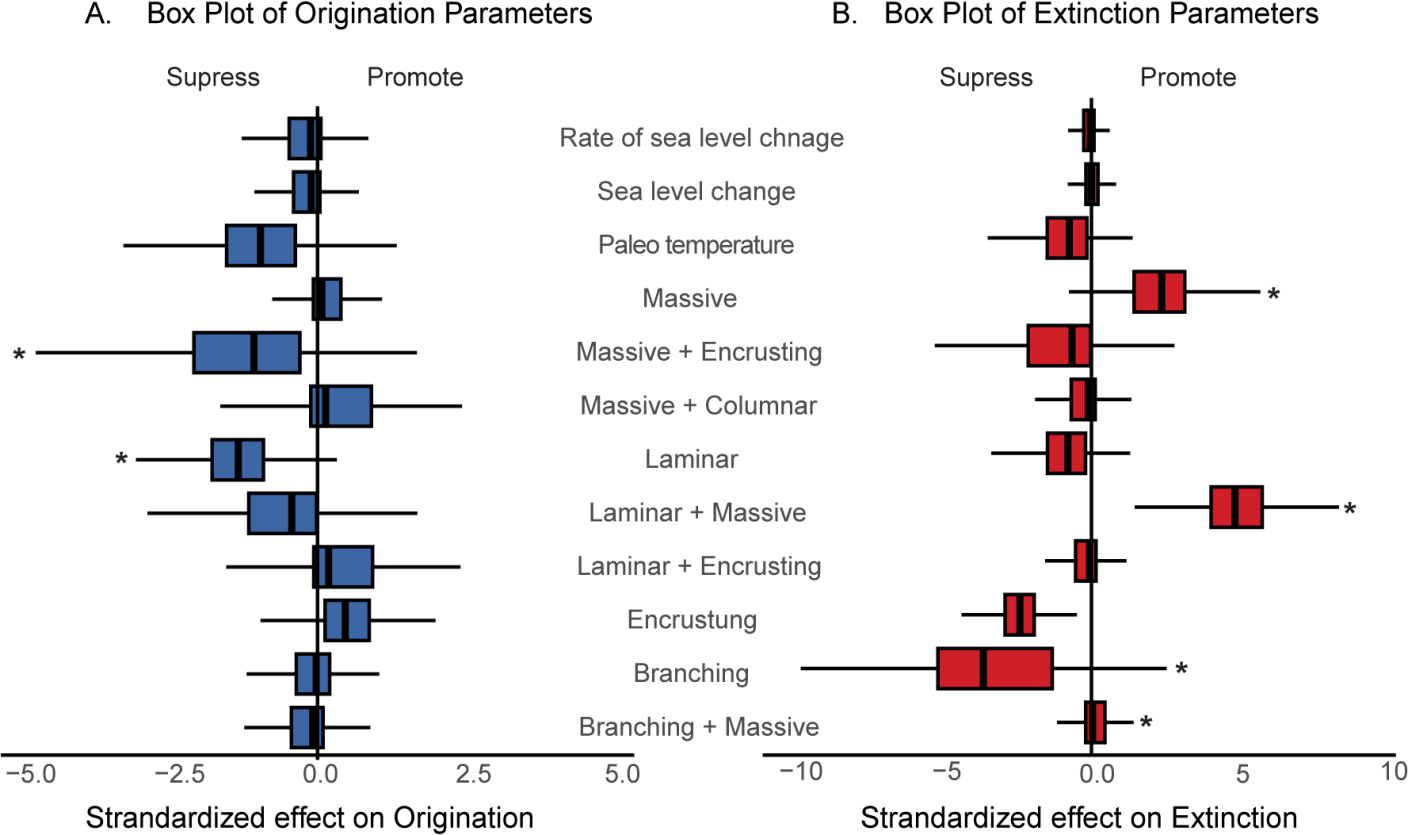
Bayesian inferences of standardized correlation parameters on origination and extinction rates with environmental and biotic factors. Boxplot showing the standardized correlation values on different predictor variables for origination (**A**) and extinction (**B**) rates across all iterations. Statistically significant relationships (median shrinkage weight > 0.6) are marked with asterisks.

**Fig 6:**
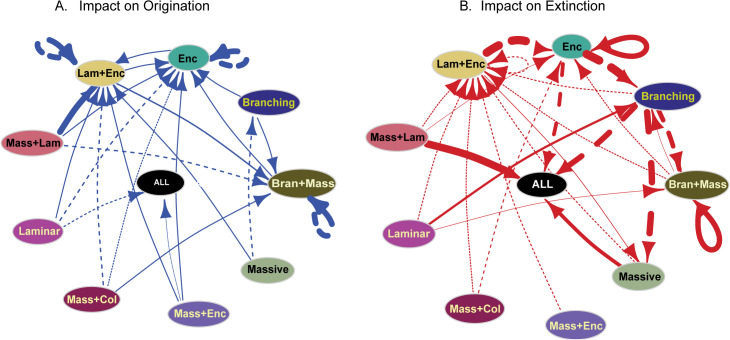
Diversity-dependent effects on origination and extinction rates within and among morpho-functional groups. This network illustrates the competitive effects imposed by each individual morpho-functional group on the origination rates (**A**) and extinction rates (**B**) of other groups. Arrows indicate the direction of effects, with arrow widths proportional to the estimated median effect size. Continuous lines denote promoting effects, while dashed lines represent suppressing effects. To minimize random variability, only relationships with shrinkage weights greater than 0.6 are included.

The multivariate birth-death model revealed the influence of species diversity within MF groups on both the origination and extinction of coral species. The model identified the effect of origination and extinction rates and the strength of the relationship for a given set of predictor variables. We noted that in addition to temperature (Gλ = −0.95, ω = 0.54) an increase in diversity within Laminar (Gλ = −1.33, ω = 0.66) and Massive+Encrusting MF group (Gλ = −1.08, ω = 0.61) results in the suppression of origination rates of coral species (Fig 5A). We found more species diversity dependent relationships on extinction rates. Increases in species diversity within Massive (Gµ = 2.3, ω = 0.81) and Massive+Laminar MF group (Gµ = 4.8, ω = 0.94) promotes extinction whereas increasing species diversity in branching MF group suppresses the extinction of coral species (Fig 5B) We extended this analysis to each MF group to determine the impact of species diversity on rate shifts within these groups. This resulted in 21 relationships for origination rates and 22 for extinction rates (see Table 3,4). We identified multiple self-suppressing relationships that were not identified in the redundancy dependence tests earlier (Fig 6). Three MF groups, i.e., Encrusting, Laminar+Encrusting, and Branching+Massive show a suppression of origination within the same group (Table 3 Fig 6A). Even though Massive and Branching groups are the most dominant in terms of diversity and occurrences, there is no impact on the rates of origination or extinction within the same MF group. The strongest positive relationship is associated with the increase in species diversity within the Massive+Laminar MF groups promoting the originations in Laminar+Encrusting group (Table 3, Fig 6A). Branching and Massive+Encrusting MF groups only had positive impacts on the origination rates (Fig 6A). But MF groups such as Massive, Laminar, Massive+Laminar, and Massive+Columnar suppress and promote the origination of species in other MF groups (Fig 6A). For extinction rates, higher diversity within Branching (Gµ = −3.6, ω = 0.94) and Encrusting (Gµ = −2.4, ω = 0.81) results in the suppression of extinctions. Branching, Laminar+Encrusting, Massive+Columnar and Massive+Encrusting only display suppression of extinction rate (Table 4). The Massive MF group makes a significant contribution to the overall suppression of extinction rate (Fig 5, Fig 6), but surprisingly we do not see any concrete evidence of high diversity within the Massive MF group selectively targeting a specific MF group (Fig 6).

**Table 3:** Impact of species diversity within morpho-functional groups on origination.

From	to	Correlation	Shrinkage weights
L	All	−1.33248	0.669656
ME	All	−1.08332	0.615118
BM	BM	−24.1181	0.904132
B	BM	1.6862	0.67007
LE	BM	1.978497	0.787759
LM	BM	−0.6609	0.661462
MC	BM	0.274483	0.664011
M	B	−0.20933	0.666715
E	E	−15.6832	0.791851
BM	E	0.628133	0.766289
B	E	0.570692	0.733812
LE	E	0.396096	0.764277
LM	E	0.550517	0.728856
L	E	−0.25011	0.81335
MC	E	−1.17905	0.72726
ME	E	0.423874	0.638579
LE	LE	−31.1284	0.851026
E	LE	0.771676	0.99599
LM	LE	18.07541	0.894578
L	LE	0.661237	0.874553
MC	LE	−0.6714	0.895276
ME	LE	0.323951	0.873917
M	LE	0.335832	0.872431

The standardized values of effect and the corresponding shrinkage weights, illustrating the impact of species diversity within one morpho-functional group on the origination rates of other MF groups. Only relationships with a shrinkage weight of 0.6 or higher are shown. The abbreviations used are as referred to in Table 1.

**Table 4:** Impact of species diversity within morpho-functional groups on extinction.

from	to	Correlation	Shrinkage weights
B	All	−3.63406	0.894682
E	All	−2.39503	0.836619
LM	All	4.821674	0.947632
M	All	2.36231	0.819421
BM	BM	3.80761	0.92624
B	BM	−3.0034	0.869491
L	BM	0.319024	0.624516
BM	B	0.235616	0.993054
E	B	−15.7154	0.735667
L	B	1.454519	0.630265
E	E	3.4872	0.675342
BM	E	−0.22467	0.612827
LE	E	−6.61624	0.660419
LM	E	0.267531	0.621728
MC	E	−0.53631	0.641083
LE	LE	−0.35987	0.899437
BM	LE	−0.23298	0.901139
B	LE	−0.30927	0.891734
LM	LE	−0.22489	0.906608
L	LE	−0.27732	0.898104
MC	LE	−0.2259	0.905618
ME	LE	−0.35064	0.901687
M	LE	−0.31221	0.895406
B	M	−4.08683	0.625097
LE	M	0.410206	0.766672

The standardized values of effect and the corresponding shrinkage weights, illustrating the impact of species diversity within one morpho-functional group on the extinction rates of other MF groups. Only relationships with a shrinkage weight of 0.6 or higher are shown. The abbreviations used are as referred to in Table 1.

## Discussion

We address the role of species diversity within morpho-functional groups, and its controls on diversity within the same group and among other co-existing groups in the same ecosystem. We evaluate this diversity dependence by exploring the impact of coral species diversity within MF groups on co-existing groups through the suppression of origination or promotion of extinction. Morpho-functional groups that we use here are the interaction of form and ecosystem function in the reef ecosystem and can be applied to other ecosystems using appropriate classifications. Species that form the MF groups have been preserved in the rock record of the Cenozoic Caribbean. Our results suggest that over-redundancy of species within a morpho-functional group does not translate to an elevated rate of extinction. However, diversity of species within different MF groups influences rates of origination and extinction within co-existing MF groups. Our study provides statistical evidence for the role of diversity within MF groups on diversification patterns of co-existing groups at an evolutionary scale.

### Diversity dependence explored through a morpho-functional approach

The impact of diversity on origination and extinction rates is often studied from a taxonomic approach, such as species diversity dependent relationships between clades or families [4,5,9] to identify the phylogenetic controls but this approach does not capture the contributions of different forms and ecosystem functions associated with different species within the same clade. In a study system such as that of scleractinian corals, novel mitochondrial examination of extant corals reveals that they are divided into complex and robust clades. Multiple subclades therein do not align with traditional family- or genus-level classifications [29–33]. For example, multiple species of the same family or even genus seem to be more closely associated with species from another family (or genus), suggesting that, with corals, the traditional taxonomic classification at family or genus level does not always capture the complex phylogenetic relationship. The main assumption that lies behind the taxonomic approach on diversity dependence is that phylogenetically closely related species compete for resources due to their shared evolutionary history [4,34–36], which leads to a net positive effect on extinction rate within the family or in others that compete for the same set of resources. Additionally, for species to compete or receive resources from others, they should co-exist and interact. Often a family level relationship is evaluated with the assumption that all the species within each family have similar ecosystem functions and interactions with co-existing organisms. There are exceptions to such a taxonomic centered approach in which diversity dependent relationships are explored based on functional and ecological characteristics [10,37]. Studies on bivalves with different life habitats (i.e., epifaunal vs infaunal) have revealed that diversity dependent diversification dynamics differ between infaunal and epifaunal bivalves; species that live above the ground (epifaunal) are found to have stronger relationships when it comes to originations and this represented an asymmetry in the patterns of extinction and origination due to diversity dependence [10] caused by variations in ecology of species. Additionally, ecology and ecosystem functions of a species are dependent on the overall morphology of the species [26,38,39], and large variations in morphology can impact the range of interactions mediated by the ecosystem functions of species [8,40,41]. Thus, a generalized assumption that all the species within a family are expected to have similar interactive relationships [4,9] would not always be true. MF grouping will help determine the role of diversity-dependent species interactions with similar forms and ecosystem functions; thus, our analyses focus on diversity patterns within morpho-functional groups, which serve as proxies for broad ecosystem-level function beyond their phylogenetic relatedness. Here we recognize that species within a single family can belong to multiple morpho-functional groups and no dominant MF group was composed of a single family. Our analysis revealed species diversity within each morpho-functional group demonstrates the range of morphological and thus functional diversity within a single family [29,42,43] even among the fossil corals. Common families such as Poritidae and Acroporidae are extremely diverse with a broad range of morphological diversity across time [44–46] and geographic zones [19,25,47]. Most poritid species at present in the Caribbean are branching with finger-like forms (except *P. astreoides* and *P. branneri*) compared to the Massive and Massive+ Encrusting ones in the Indo- Pacific [48]. The extant species within Acroporidae in the Caribbean are branching forms and belong to a single genus (*Acropora*). Whereas the Indo- Pacific has a greater range of generic and morphologic diversity, we find *Montipora*, *Acropora* and *Alvepora* in the Caribbean belonging to Branching, Massive, Laminar, Laminar + Foliaceous and Foliaceus MF groups. With such a wide range of morphological diversity within a single family (e.g., here the family Acroporidae), it is likely that interactions of different species within the same family are not uniform.

### Absence of redundancy related impacts

Biotic interactive relationships such as resource competition have the ability to shape macro evolutionary patterns [49,50]. During a period of limited resources, over redundancy can mediate diversity patterns through time [1,51,52]. Multiple species with similar morphologies that shares ecosystem functions and habitats lead to functional redundancy [53]. This increases competition among redundant species through resource limitation [37,54]. For coral species with similar morphology and ecosystem functions, grouped as a morpho-functional (MF) group, we expected an increased rate of extinction due to over-redundancy. However, results from this work suggest otherwise. Even among the MF groups with the highest redundancy, Massive and Branching, we failed to observe any significant negative diversity dependence.

### Impacts of diversity within morpho-functional groups

Our study indicates that, in addition to environmental factors, there is compelling evidence for diversity dependent interactive relationships to influence the expansion and elimination of different coral morpho-functional groups and by inference, the stability of the reef ecosystem. Sedentary organisms such as corals can show aggression and competition with the co-existing species [55,56]. Such competition can occur within the same coral species or between species that have similar or different functional roles with similar niche requirements [49,56–62]. Our results show an interconnected array of networks across different groups, such as increasing diversity within the Laminar MF group promoting the extinction of the Branching MF group. This negative relationship between the groups could be associated with competition for resources. It is established through empirical studies that fast-growing corals compete for space and could outcompete the other groups, providing them little opportunity to proliferate [55,63]. Here, though species in both Laminar and Branching groups show fast growth, they have two distinct directions of growth. The high degree of lateral growth of the Laminar MF groups makes it efficient at photo-adaptability and could prevent the species from other co-existing groups from receiving adequate sunlight [26,64] and thus limit their proliferation potential. The species within Branching MF groups tend to focus on vertical growth and this growth could get stunted if it co-exists with Laminar corals due to lack of sunlight. We also notice that the interactions or relationships that we find across different MF groups are usually affected by the reef zones they occupy. While we found that the increasing species diversity within the Laminar group impacts the Branching group, exhibited through the promotion of extinction rate of the Branching, we also observed that a higher diversity within the Branching MF group contributed to the suppression of extinction of species within the Massive MF group. These chains of interactive relationships between the diversity of species within various MF groups demonstrate a cumulative array of connections. The shifts in diversity within a single MF group can impact other groups even though there is no direct link between the MF groups, thus resulting in a domino effect (Fig 6).

Increased diversity within a group does not always translate to competition or have a net negative effect that leads to the demise of species or groups. Increasing diversity can also facilitate [1] the proliferation of other groups. An enhanced biodiversity of reef corals promotes primary productivity in the reef ecosystem through increased availability of tissues for residential photosynthetically active algae [65]. In addition to promoting reef performance, intermediate to high coral species diversity supports a faster and efficient recovery process post decline in population (e.g., the decline in population could be due to bleaching or physical destruction after a storm), and restoration efforts tend to be more successful when corals are transplanted in a habitat with higher diversity [66,67]. Here we discovered that change in species diversity within multiple MF groups promote origination and suppress the extinction rate for others. A prominent example being the impact of increased diversity within the Branching MF group on co-existing groups, for which the high diversity leads to suppressed extinction rates across multiple MF groups including the Massive MF group. Species within the Branching group are usually fast growing with a higher rate of calcification [68], and they also tend to be more structurally complex with increased fractal dimension [17] which creates microhabitats for the facilitation of larval dispersion [69] and promotes the survival potential for other species. These ecosystem functions associated with the branching group are manifested in positive impacts on coexisting species through the suppression of extinction at an evolutionary timescale.

### Considerations and future directions

Even though our study provides evidence for diversity dependence across multiple MF groups while identifying the lack of redundancy-related impacts within a given MF group, these results come with multiple assumptions and are sensitive to the selected predictor variables. Considerations of carbonate compensation depth remain well below the habitat range of shallow water, zooxanthellate corals considered in this study. Deep-sea CCD shoaling primarily reflects deep-ocean carbonate dissolution rather than direct control on shallow-water calcification [70,71]. Future research would need to investigate the influence of low pH values in deep water and the lag time to pH-induced dissolution in shallow water corals in extinction studies. In an another consideration stable reef ecosystem is composed of multiple elements in addition to shallow water corals, and these include reef fishes, macro algae and encrusting sponges [24,72]. We not only observe biotic interactions among coral species that form the MF groups, but also other co-existing taxonomic groups [56,73–75], and shifts in composition of other non-coral taxa such as herbivorous fishes [76,77] and macro-algal [68,78,79] populations all of which strongly impact coral diversity and population size [73,76,80–82]. But research presented here focuses exclusively on the impact of coral species diversity and morphology on themselves, and this presents a major step in understanding the role of co-existing organisms and their morphology in shaping ecosystem diversity through geologic time and into the future. Additionally, usage of a functional grouping approach comes with its own set of challenges. FG (Functional Group) classification is often based on subjective characters and even if we are to use measurable traits, they tend to be continuous, and the choice of boundaries can be questionable [83–85]. Additionally, such a classification can be impacted by species identifications. Taxonomic identifications and growth form assignments in this study were based on published literature and expert-validated compilations, and we applied consistent criteria across all species and groups. Moreover, here we have tried to quantify the descriptive character such as growth form using a binary classification to best address our hypothesis. Another challenge is associated with the choice of geographic range of the study. Here we focus on a single biogeographic region, and this represents only a subset of coral species and thus a subset of theoretically possible MF groups. Wider geographic range leads to an increased number of MF groups and alters the species diversity within each group, and this can be manifested into interactive patterns that are not ecologically plausible. A global study would not be efficient in capturing the relationship between provincial species, and one might get a false positive correlative relationship between two groups of species that might not even coexist. Meanwhile, our regional approach minimizes geographic bias, improving the credibility of our results.

The complex habitat structure created by different organisms controls community organization in an ecosystem by determining which species persist [35,86]. Scleractinian corals are one of the primary ecosystem engineers responsible for creating biogenic habitat structures in the marine ecosystem, for they are not only capable of being ecosystem engineers that are autogenic (creating new habitats) but also allogeneic (modifying the current ecosystem) [87,88]. The role and contribution of corals in shaping the habitat structure and architectural complexity of a reef ecosystem are driven by their overall morphology [89]. At a macroevolutionary scale while the changes in climate control the biodiversity, species diversity dependence also remains a prominent cause [3,4,10,90] and this work demonstrates the role of ecologically significant coral MF groups and the species diversity within each group in controlling the diversity patterns across co-existing groups.

### Coda

An evolutionary scale study such as this acts as a framework to show the significance of species diversity on the long-term maintenance of morpho-functional groups and ecosystem functions. A non-taxonomic centric approach that focuses on the morphology and ecology of coral species offers a novel perspective on the shift in species composition and thus offers a chance to explore the habitat structure through time, while identifying the intensity of originations and extinctions of species that belong to a specific MF group and the role of changing species diversity within one group on another. While the diversity-dependent diversification hypothesis is well established, a macro-evolutionary framework using MF groups highlights the non-uniform impact of morphology-mediated diversity dependence on co-existing species and introduces a novel perspective for dissecting diversity dependent relationships.

## Methods

We test species diversity dependence using hermatypic zooxanthellae bearing scleractinian corals. We create morpho-functional groups based on the growth form data and analyze the species diversity dependence across different morpho-functional groups.

### Occurrence data for corals

**Fig 7:**
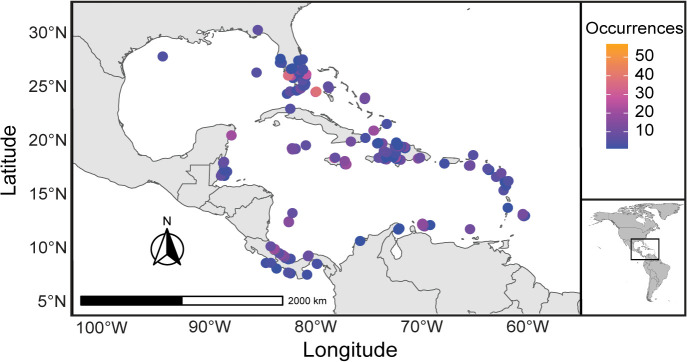
Map of the study area in the Caribbean. Present day map of the study area with Paleocene through Pleistocene occurrences of fossil scleractinian corals, with lighter color indicating higher occurrence frequences at a given location. The inset map provides a broader geographic context, with a square box marking the Caribbean study area.

For this research we downloaded 26,289 records for global scleractinian occurrences from Paleocene through Pleistocene from the Paleobiology database (https://paleobiodb.org/classic; data accessed February 2023- June 2023). Shortlisting Caribbean occurrences for the geographic boundaries of longitude −98.3424 to −55.9778 and latitude 7.4 to 33.514, here called the Caribbean ecoregion (Fig 7), resulted in 7300 occurrences. Records that exhibited taxonomic inconsistencies such as uncertain genus or species and those identified as sp., aff., were removed from further analysis. We cross-verified and updated the taxonomy with currently accepted scientific names using the World Register of Marine Species (WORMS https://www.marinespecies.org) for each coral species and again removed any species with dubious nomenclature and occurrence. All potential non-reef building, azooxanthellate and deep-water coral species were removed, and further analysis focused only on hermatypic and zooxanthellate corals. This resulted in 6116 occurrences represented by 271 unique species (Fig 7).

### Growth form data transferred to morpho-functional groups

We gathered the growth from data for the species from Coral Trait Database [91], Corals of the World [19], Neogene Marine Biota of Tropical America (NMITA) [92], Ancient coral trait database [93], Corallosphere (https://www.corallosphere.org/) and the Treatise of Invertebrate paleontology [94]. We used Veron’s standard growth forms of solitary, laminar, foliaceous, massive, branching, encrusting, and columnar [19] to categorize coral species forms, based on the species characteristics. Species which only displayed solitary morphology were then excluded and we proceeded with the species which exhibit colonial growth forms (Fig 1). This resulted in 5973 occurrences represented by 250 species. Since coral species displayed ecophenotypic variations, we considered all growth forms that a species display. Growth form is treated as a binary variable and if a species displayed a given growth form it was given a score of 1, if not 0. Each unique combination that resulted from the binary scoring of six growth forms was categorized into a distinct MF group (The data and code used in this study can be found at https://doi.org/10.6084/m9.figshare.30086608).

### PyRate analysis

We performed the species diversity dependence analysis on scleractinian coral species diversity within morpho-functional groups using PyRate [27]. PyRate is an algorithm developed and executed using the python program, it uses a Bayesian framework to compute the diversification parameters. The datasets are analyzed using a birth-death model to estimate i) origination time (*Ts*), ii) and extinction time (*Te*) for each species, iii) rates of origination(λ) and iv) extinction (µ) and v) the frequency and intensity of rate shifts within each MF group. The results from the model are estimated throughout the study interval at every 0.1 Myr interval and the results are used to identify the uniformity of species diversity and rates of origination and extinction within each MF group. The rates and diversity trajectories (range through time plots) are reported at 0.1 million year (Myr) increments. We choose this value as a compromise between temporal resolution and the uncertainty in fossil ages and preservation. We further estimated the preservation parameters (q) per million years, which would assist in calibrating the occurrence data. Prior to running the algorithm, the -PPmodeltest option was used to identify the best preservation model to avoid preservation bias based on the lowest AIC score. This option compares different preservation models available, such as homogeneous Poisson process (HPP), non-homogeneous Poisson process (NHPP) and time variable Poisson process (TPP, analysis performed with -q). The test revealed the time variable Poisson process (TPP) to be the best option for the given dataset (S1 Table).

For the 17 MF groups that were identified in this study, we used the PyRate algorithm to employ a reversible jump Markov chain Monte Carlo (rjMCMC) analysis to estimate the timing and rates of origination and extinction. To reduce any inconsistency with the time of occurrence (i.e., fossil ages) of each species we generated 10 randomized replicates for each MF group by resampling age estimates for each observation. We ran the algorithm for 5 million generations of rjMCMC with a sampling frequency of 5000. Outputs included 1000 observations of posterior and prior distributions, estimations of *Ts* and *Te*, and rates of origination and extinction for each replicate. The origination and extinction rates and the frequency of rates shifts were visualized using the -plotRJ function using the outputs from the rjMCMC PyRate analysis, and the -ltt function was used to plot the species diversity through time (we used 0.1 Myr increments, using a coarser time bin (E.g. 1 Myr) would potentially mask rate shift pulses). The posterior estimates from the results were visualized using the Tracer application [95] to ensure convergence of values, and the initial 100 observations (10 percent) were removed as burn-in. The outputs from each of the replicates were then combined, yielding 10 sets of values for *Ts* (speciation time) and *Te* (extinction time). Out of the 900 values within each set 100 values were randomly selected to create the final set with 1000 values for *Ts* and *Te*. The selected values were then used to extract the corresponding rates of origination and extinction within each group. The resampled set was then used for all analysis that followed.

### Functional indices

Functional indices were computed and tested for significance prior to establishing the impact of redundancy related diversity dependence on extinction. Functional richness is represented by the total number of morpho-functional groups created from the distinct combinations of growth form data. Functional redundancy (FR) and functional over-redundancy (FOR) [96] were computed to estimate the skewness of species diversity data and significance of the skewness observed. Given, FG is the total number of morpho-functional groups, S the total number of species, and ${\text{n}}_{\text{i}}$ the number of species in each functional group,


FR=S/FG (1)

$$\text{FOR}=\;\frac{\sum_{\text{i}=\text{1}}^{\text{FG}}\left[\text{max}\left({\text{n}}_{\text{i}},\text{FR}\right)-\text{FR}\right]}{\text{S}}$$ (2)

The observed FR and FOR were then compared with a null model to test the significance of the observation, since these indices are impacted by the number of species and functional groups. We ran 9999 simulations with total number of species and functional groups constant, to test whether the skewness is expected in the distribution. We used a bilateral t test with 95% confidence interval to assess the significance. These calculations were repeated for each epoch studied here.

### Impact of redundancy

To assess whether species redundancy within a morpho-functional group promotes extinction within that group, we used the multi-trait-dependent extinction model (MTE) within PyRate [37]. The analysis is used to differentiate whether the extinctions are associated with the growth form of the species or are a result of species diversity within a MF group, or both. The Te (extinction time) estimates from the above rjMCMC (reverse jump) estimation is used here to perform the MTE analysis  .

**Table 5:** Log calibrated categorization of species diversity.

Number of species (S)	Log calibration (log (S))	Category
1	0	1
< 4	<0.5	2
< 10	<1	3
< 32	<1.5	4
> = 32	>1.5	5

An arbitrary log calibration is used to categorize the number of species within morpho-functional groups. MF group is assigned a category value between 1–5 based on the log values of the number of species found within each group.

Since the redundancy values (i.e., number of species occupying a MF group) ranged from 1 to 123, the values were log transformed to create arbitrary categorical variables that could be used in the prediction. The log transformation resulted in 5 categories (1–5 used as a categorical predictor) as depicted in Table 5.

We ran the analysis for 1.5 million MCMC iterations for the entire dataset and repeated the analysis for 10 replicates to ensure validity of the results. For the MTE model, the computed extinction rate ($\mu_{\text{i}}$) is parameterized as a function of the mean rate ($\mu_{\text{0}}$) and Dirichlet-distributed multiplier, which here encompasses the growth form and redundancy dependence. The model is built so that it can include multiple features to affect extinction rates, and here the growth form classification and redundancy associated with the group that a species belongs to. The model is implemented in a Bayesian framework to compute the parameter values by using an rjMCMC algorithm to calculate the posterior values for the parameters. The model uses Bayesian shrinkage and Bayesian variable selection algorithm [4,37] to reduce over-parameterization and false positives resulting from incorporation of non-influential features.

### Climate data

In the species diversity dependence analysis, we used paleo sea surface temperatures (SST) and sea–level fluctuations as predictor variables, along with species diversity within MF groups. We used these predictor variables because coral diversification is sensitive to temperature and sea level changes [44,97–99]. While corals are also found to be sensitive to atmospheric CO₂ and ocean carbonate chemistry [97], studies have found that CO₂ changes during the early Cenozoic are strongly coupled to SST [100] and to changes in surface-water carbonate chemistry [101]. Including known correlative variables such as SST, CO₂ concentration, and pH introduces the risk of redundancy and multicollinearity that can lead to model overfitting. We used global mean paleotemperature data estimated from benthic foraminifera isotope records that uniformly span the 66 million year history of the Cenozoic [102]. Eustatic sea-level records for the Cenozoic were obtained from Miller et. al [103] who extracted the records by back-stripping stratigraphic data from New Jersey coastal plain core holes. We extracted data for every 0.1 million year time interval for the environmental parameters and standardized the values between 0 and 1 to maintain uniformity between predictors.

### Impact of diversity

We used the MBD (multivariate birth death) model within PyRate [27,104] to analyze the role of species diversity within a morpho-functional group on another along with the afore mentioned climate variables. This model computes the strength of diversity dependence on the origination and extinction of species within various MF groups. Out of the 17 MF groups identified in this study, only nine were deemed dominant with a species diversity representation by minimum five species and 70 occurrences per group and were selected for the MBD analysis. The impact of diversity within different MF groups was analyzed by evaluating its correlation with respect to rates of origination and extinction. The original multi-clade diversity dependence model (MCDD) was adapted to assess the impact of positive and negative effects of species diversity within a MF group on the extinction or origination of species within itself and others. The model was recently updated with Horseshoe prior to incorporating the impacts of overparameterization, and it was further modified to add possibilities of nonlinear relationships such as exponential [104]. Here we used the latest model called the Multivariate Birth Death (MBD) model, to assess the role of diversity within different morpho-functional groups. The *Ts* and *Te* extracted from previous analysis are used as the input for the MBD. The relative diversities scaled between 0 and 1 were used as the predictors for the diversity dependent model. We then ran the model for 15 million iterations with a sampling frequency of 15000 for the 10 replicates. The results of the MBD analysis include the posterior values, Gλ (impact on origination) and Gµ (impact on extinction) and the shrinkage weights (ω) for each predictor. The values of Gλ and Gµ signify an increase in origination and extinction rate variation above the baseline rates and shrinkage weights describe the significance of Gλ and Gµ values, and the values ranges from 0–1. When the shrinkage weights are less than 0.5, the observed relationships may manifest background noise. To visualize and summarize our results we extracted the median values and 95% confidence intervals of the outcomes after removing the first 20 percent of the outcome as burn-in. This was repeated for the whole dataset as well as for each of the MF groups. Results with a shrinkage weight of 0.6 or more from the analysis for each of the groups was combined to summarize diversity dependence across all the dominant MF groups.

## Supporting information

S1 Fig Diversity shifts and rate shifts within the Massive group. (A) Change in origination rates over time. (B) Frequency of origination rate shifts. (C) Change in extinction rates over time. (D) Frequency of extinction rate shifts. (E) Net diversification rates, and (F) Range through time plot for Massive group. Solid lines indicate mean posterior rates and shaded areas show 95% CI.
(TIF)

S2 Fig Diversity shifts and rate shifts within the Branching group. (A) Change in origination rates over time. (B) Frequency of origination rate shifts. (C) Change in extinction rates over time. (D) Frequency of extinction rate shifts. (E) Net diversification rates, and (F) Range through time plot for Branching group. Solid lines indicate mean posterior rates and shaded areas show 95% CI.
(TIF)

S3 Fig Diversity shifts and rate shifts within the Massive + Encrusting group. (A) Change in origination rates over time. (B) Frequency of origination rate shifts. (C) Change in extinction rates over time. (D) Frequency of extinction rate shifts. (E) Net diversification rates, and (F) Range through time plot for Massive + Encrusting group. Solid lines indicate mean posterior rates and shaded areas show 95% CI.
(TIF)

S4 Fig Diversity shifts and rate shifts within the Massive + Laminar group. (A) Change in origination rates over time. (B) Frequency of origination rate shifts. (C) Change in extinction rates over time. (D) Frequency of extinction rate shifts. (E) Net diversification rates, and (F) Range through time plot for Massive + Laminar group. Solid lines indicate mean posterior rates and shaded areas show 95% CI.
(TIF)

S5 Fig Diversity shifts and rate shifts within the Encrusting group. (A) Change in origination rates over time. (B) Frequency of origination rate shifts. (C) Change in extinction rates over time. (D) Frequency of extinction rate shifts. (E) Net diversification rates, and (F) Range through time plot for Encrusting group. Solid lines indicate mean posterior rates and shaded areas show 95% CI.
(TIF)

S6 Fig Diversity shifts and rate shifts within the Laminar group. (A) Change in origination rates over time. (B) Frequency of origination rate shifts. (C) Change in extinction rates over time. (D) Frequency of extinction rate shifts. (E) Net diversification rates, and (F) Range through time plot for Laminar group. Solid lines indicate mean posterior rates and shaded areas show 95% CI.
(TIF)

S7 Fig Diversity shifts and rate shifts within the Branching + Massive group. (A) Change in origination rates over time. (B) Frequency of origination rate shifts. (C) Change in extinction rates over time. (D) Frequency of extinction rate shifts. (E) Net diversification rates, and (F) Range through time plot for Branching + Massive group. Solid lines indicate mean posterior rates and shaded areas show 95% CI.
(TIF)

S8 Fig Diversity shifts and rate shifts within the Massive + Columnar group. (A) Change in origination rates over time. (B) Frequency of origination rate shifts. (C) Change in extinction rates over time. (D) Frequency of extinction rate shifts. (E) Net diversification rates, and (F) Range through time plot for Massive + Columnar group. Solid lines indicate mean posterior rates and shaded areas show 95% CI.
(TIF)

S9 Fig Diversity shifts and rate shifts within the Laminar + Encrusting group. (A) Change in origination rates over time. (B) Frequency of origination rate shifts. (C) Change in extinction rates over time. (D) Frequency of extinction rate shifts. (E) Net diversification rates, and (F) Range through time plot for Laminar + Encrusting group. Solid lines indicate mean posterior rates and shaded areas show 95% CI.
(TIF)

S10 Fig Impact of Growth Form, Redundancy, and Colony Form on Extinction Rates. (A) The relative effects of growth form, redundancy, and colony form on extinction, calculated from the posterior probability of inclusion for each pair of coral traits in the multi-trait-dependent extinction analyses. Dashed lines indicate thresholds corresponding to the log Bayes factor, with the bottom, middle, and top lines representing positive, strong, and very strong statistical support, respectively. The relative impact of individual components of the (B) traits and (C) redundancy on extinction rates, with the dotted line indicating expected values under a null model. The p-values for the observed relationships are displayed at the top of each graph.
(TIF)

S1 Table Preservation model test results. The -PPmodeltest function was used to test for the best preservation model for the given dataset. The 3 models considered here includes homogeneous Poisson process (HPP), time-variable Poisson process (TPP) and non-homogeneous Poisson process of preservation (NHPP). The best model was selected based on the lowest AIC score.
(XLSX)
