# Supplementary figures and images for: Species diversity within morpho-functional groups drives diversity dependence in reef ecosystems
Source: PLoS One. 2025 Dec 17;20(12):e0338441. doi: 10.1371/journal.pone.0338441 (PMC12711069; doi:10.1371/journal.pone.0338441)

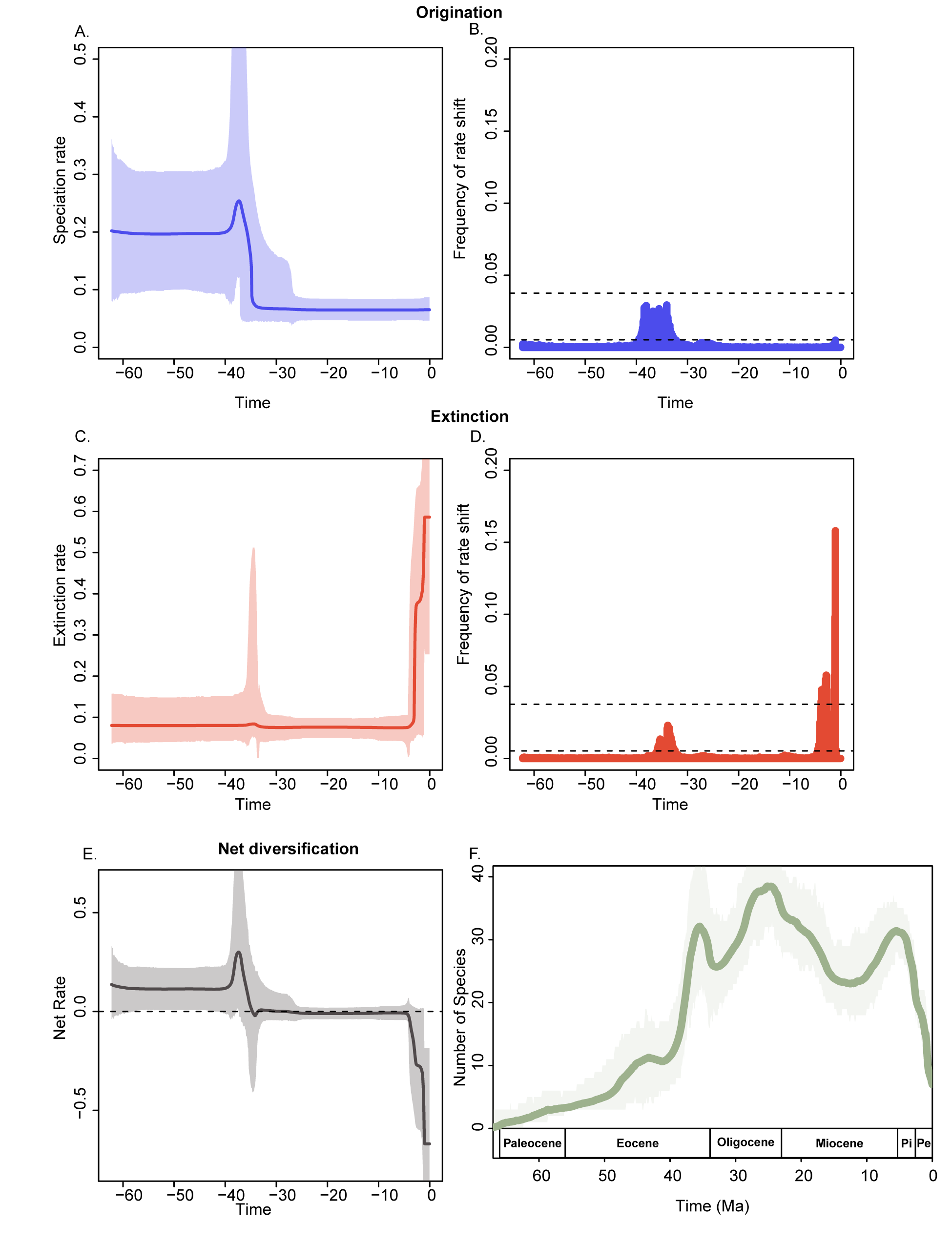

Supplement: S1 Fig — (A) Change in origination rates over time. (B) Frequency of origination rate shifts. (C) Change in extinction rates over time. (D) Frequency of extinction rate shifts. (E) Net diversification rates, and (F) Range through time plot for Massive group. Solid lines indicate mean posterior rates and shaded areas show 95% CI. (TIF) [file pone.0338441.s001.tif]

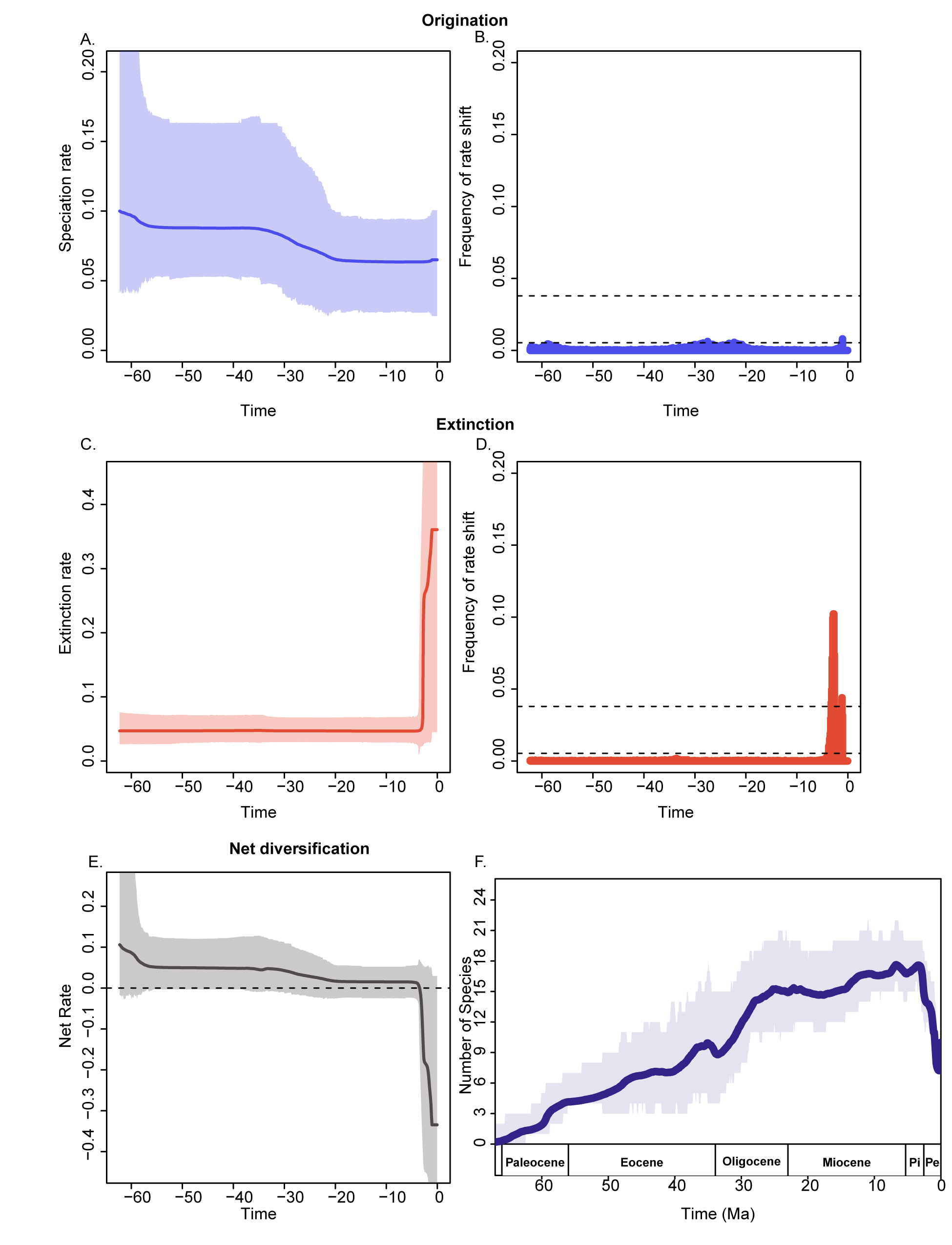

Supplement: S2 Fig — (A) Change in origination rates over time. (B) Frequency of origination rate shifts. (C) Change in extinction rates over time. (D) Frequency of extinction rate shifts. (E) Net diversification rates, and (F) Range through time plot for Branching group. Solid lines indicate mean posterior rates and shaded areas show 95% CI. (TIF) [file pone.0338441.s002.tif]

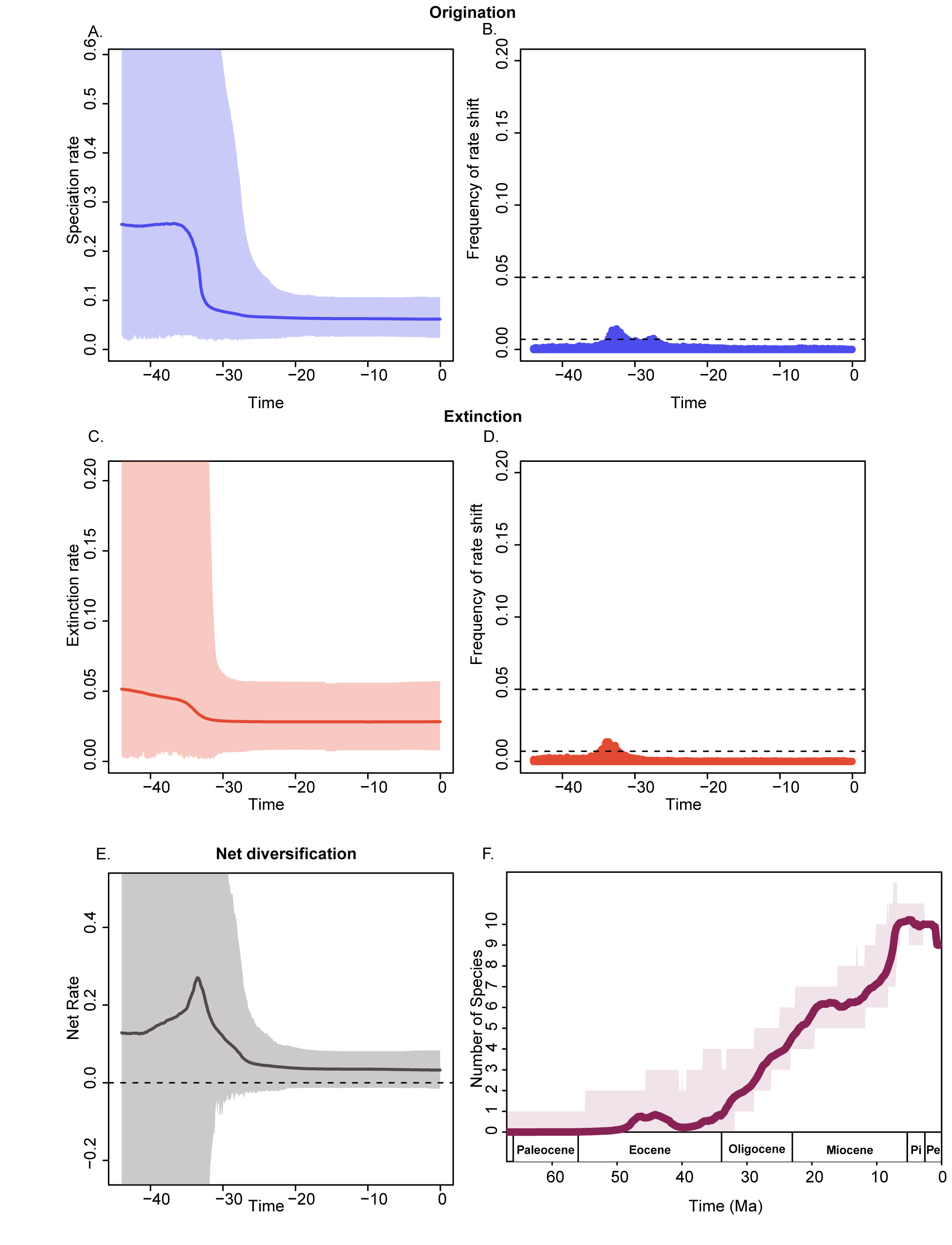

Supplement: S3 Fig — (A) Change in origination rates over time. (B) Frequency of origination rate shifts. (C) Change in extinction rates over time. (D) Frequency of extinction rate shifts. (E) Net diversification rates, and (F) Range through time plot for Massive + Encrusting group. Solid lines indicate mean posterior rates and shaded areas show 95% CI. (TIF) [file pone.0338441.s003.tif]

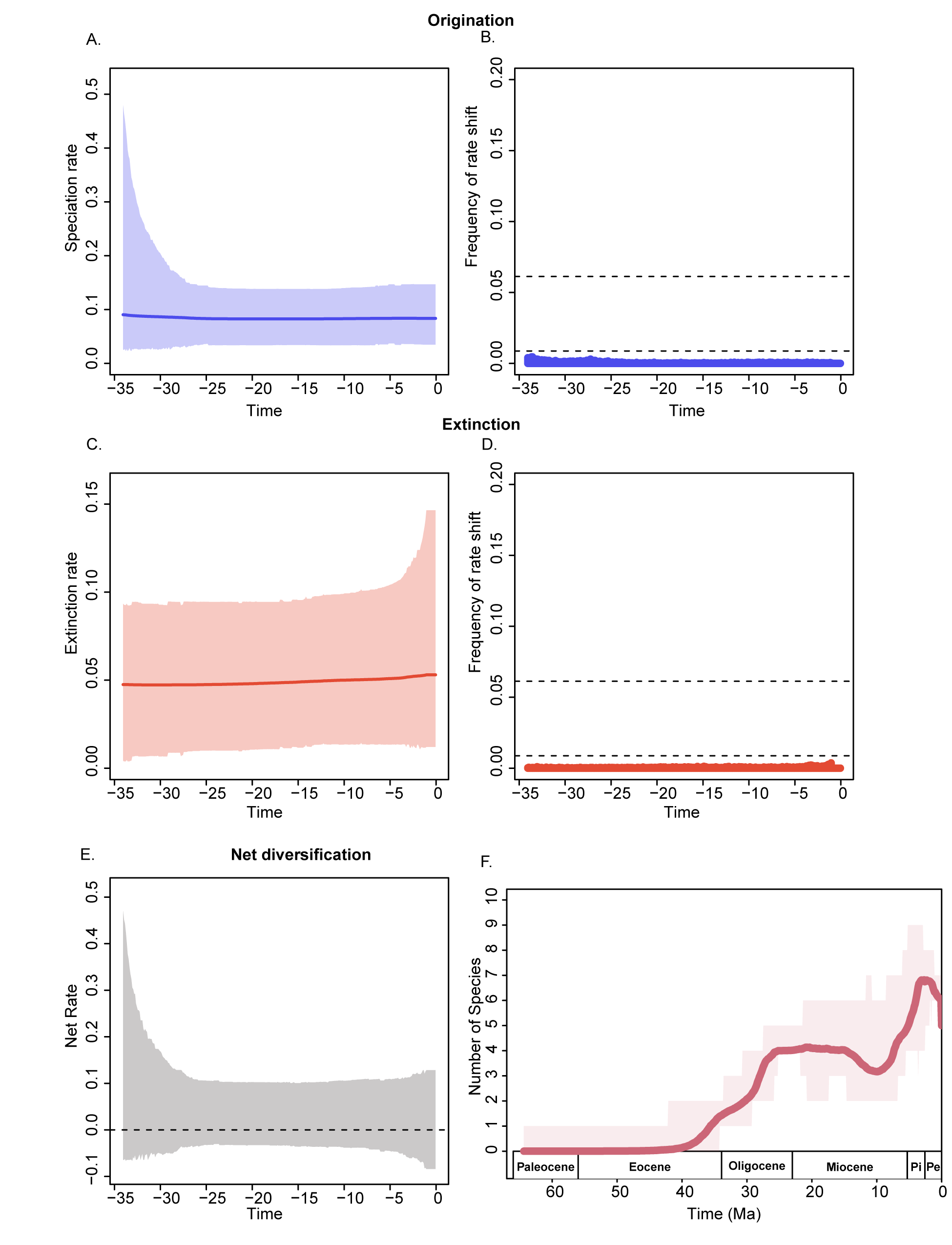

Supplement: S4 Fig — (A) Change in origination rates over time. (B) Frequency of origination rate shifts. (C) Change in extinction rates over time. (D) Frequency of extinction rate shifts. (E) Net diversification rates, and (F) Range through time plot for Massive + Laminar group. Solid lines indicate mean posterior rates and shaded areas show 95% CI. (TIF) [file pone.0338441.s004.tif]

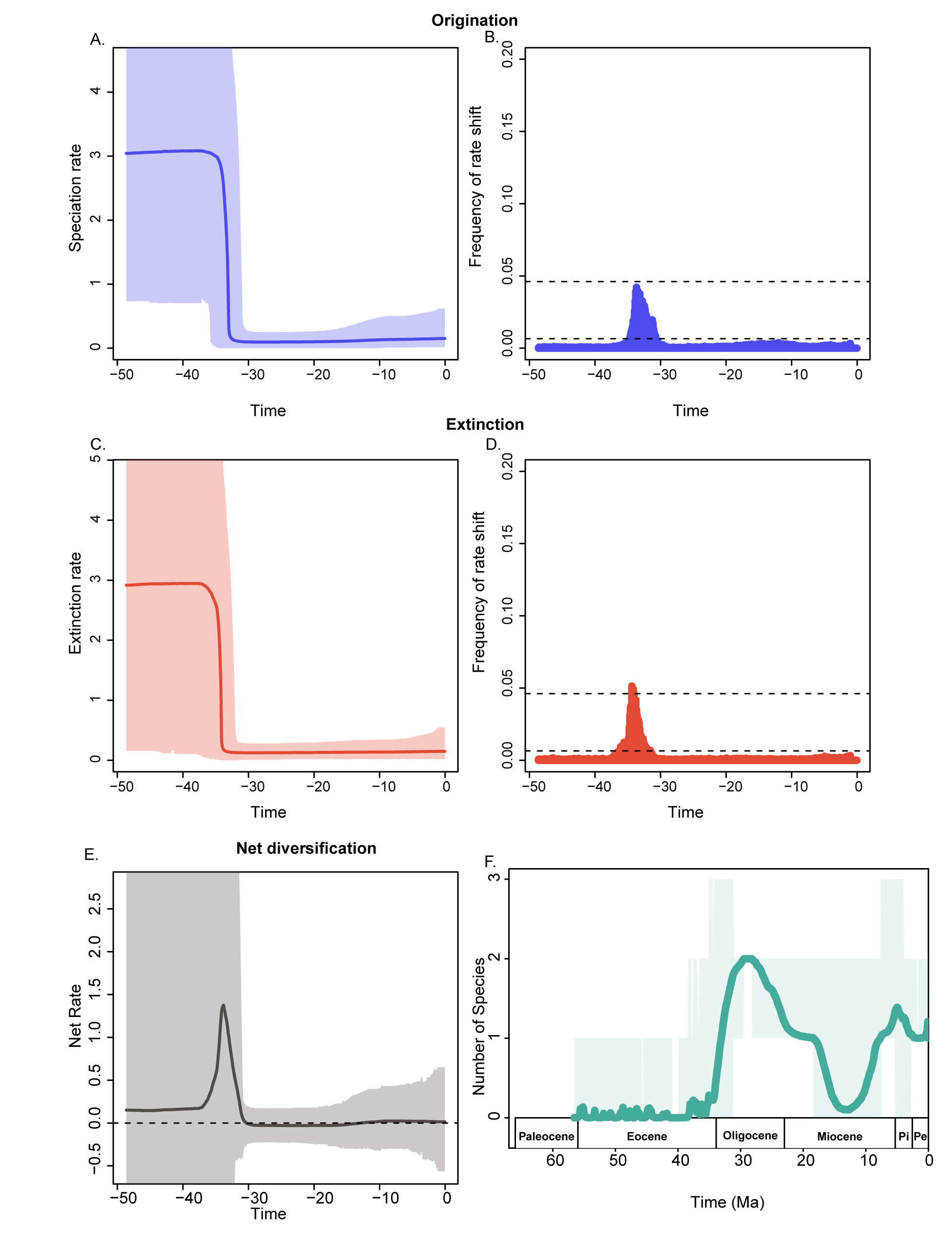

Supplement: S5 Fig — (A) Change in origination rates over time. (B) Frequency of origination rate shifts. (C) Change in extinction rates over time. (D) Frequency of extinction rate shifts. (E) Net diversification rates, and (F) Range through time plot for Encrusting group. Solid lines indicate mean posterior rates and shaded areas show 95% CI. (TIF) [file pone.0338441.s005.tif]

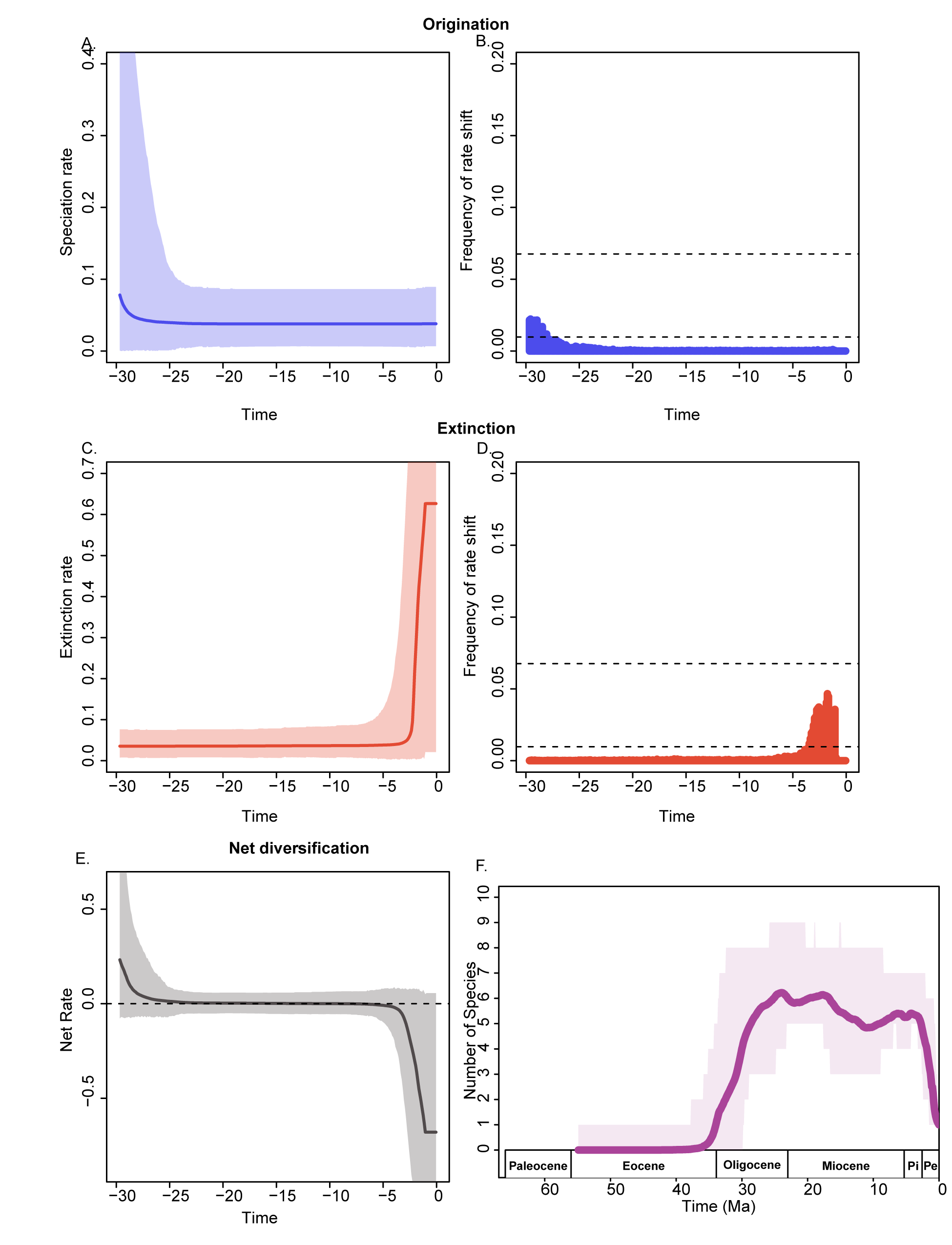

Supplement: S6 Fig — (A) Change in origination rates over time. (B) Frequency of origination rate shifts. (C) Change in extinction rates over time. (D) Frequency of extinction rate shifts. (E) Net diversification rates, and (F) Range through time plot for Laminar group. Solid lines indicate mean posterior rates and shaded areas show 95% CI. (TIF) [file pone.0338441.s006.tif]

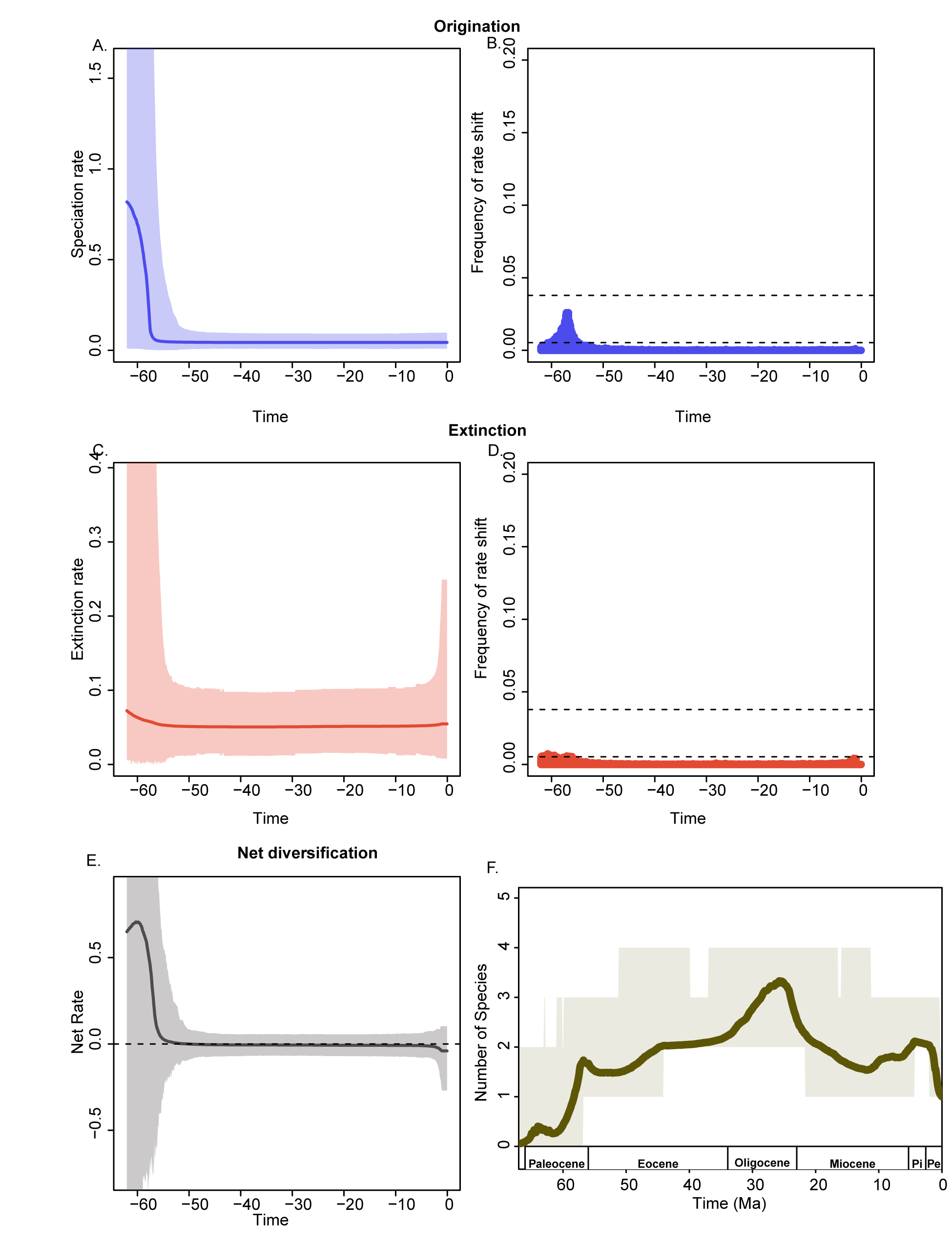

Supplement: S7 Fig — (A) Change in origination rates over time. (B) Frequency of origination rate shifts. (C) Change in extinction rates over time. (D) Frequency of extinction rate shifts. (E) Net diversification rates, and (F) Range through time plot for Branching + Massive group. Solid lines indicate mean posterior rates and shaded areas show 95% CI. (TIF) [file pone.0338441.s007.tif]

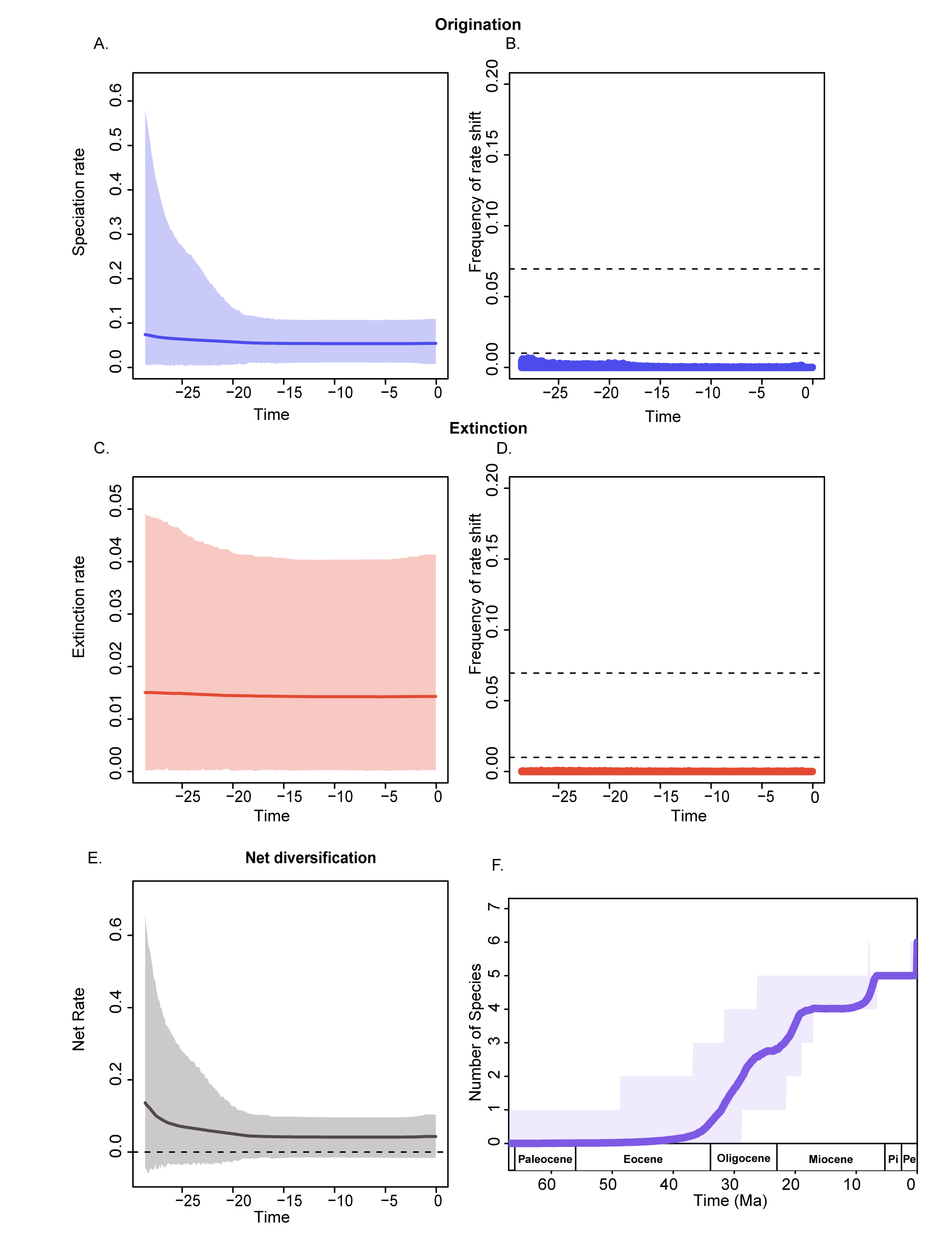

Supplement: S8 Fig — (A) Change in origination rates over time. (B) Frequency of origination rate shifts. (C) Change in extinction rates over time. (D) Frequency of extinction rate shifts. (E) Net diversification rates, and (F) Range through time plot for Massive + Columnar group. Solid lines indicate mean posterior rates and shaded areas show 95% CI. (TIF) [file pone.0338441.s008.tif]

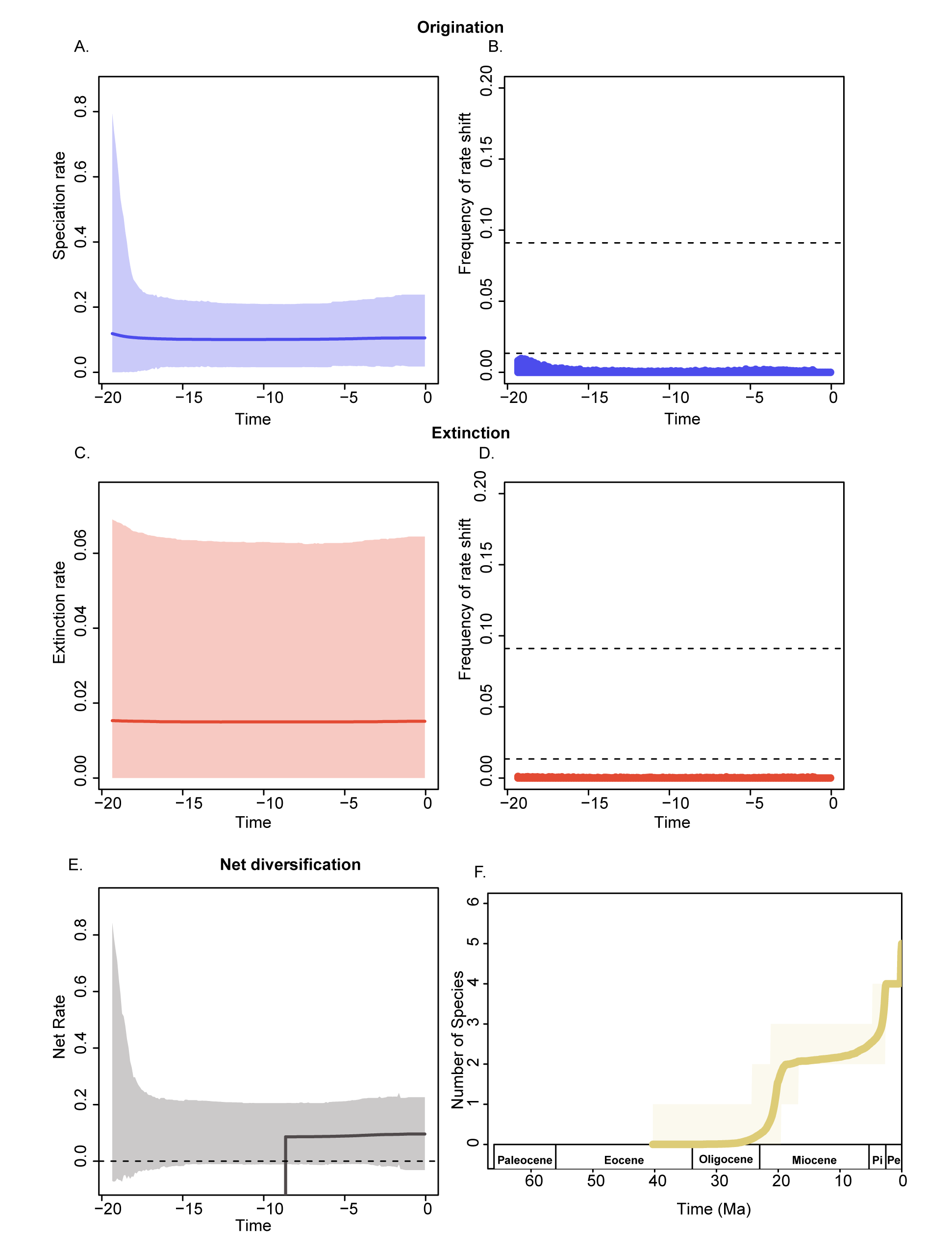

Supplement: S9 Fig — (A) Change in origination rates over time. (B) Frequency of origination rate shifts. (C) Change in extinction rates over time. (D) Frequency of extinction rate shifts. (E) Net diversification rates, and (F) Range through time plot for Laminar + Encrusting group. Solid lines indicate mean posterior rates and shaded areas show 95% CI. (TIF) [file pone.0338441.s009.tif]

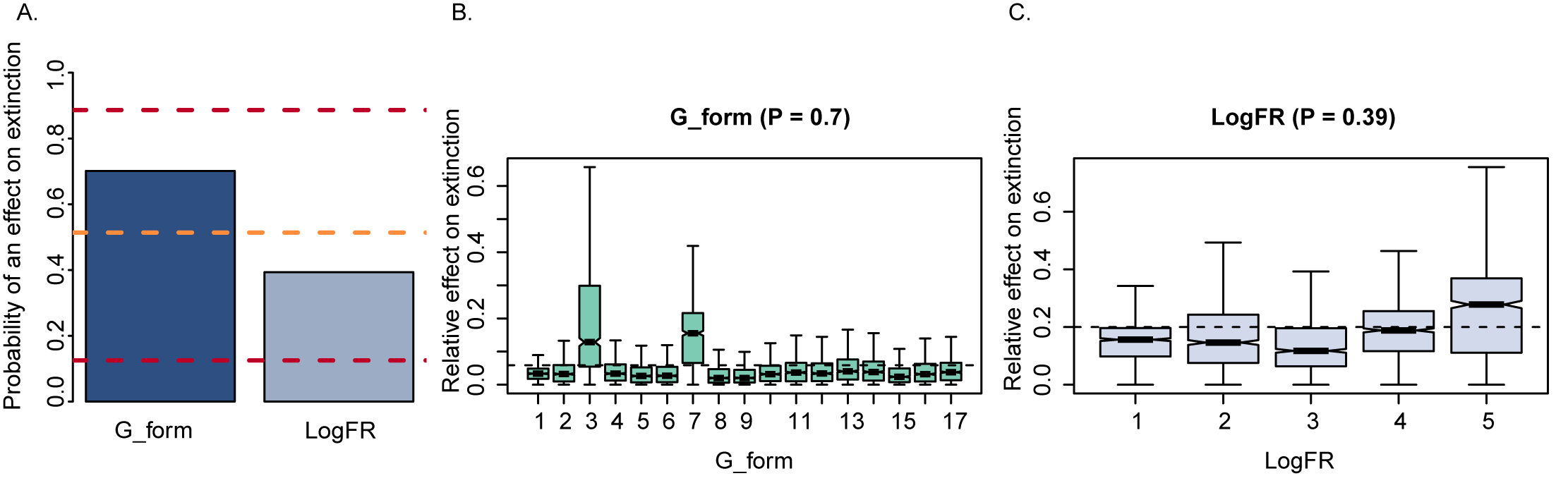

Supplement: S10 Fig — (A) The relative effects of growth form, redundancy, and colony form on extinction, calculated from the posterior probability of inclusion for each pair of coral traits in the multi-trait-dependent extinction analyses. Dashed lines indicate thresholds corresponding to the log Bayes factor, with the bottom, middle, and top lines representing positive, strong, and very strong statistical support, respectively. The relative impact of individual components of the (B) traits and (C) redundancy on extinction rates, with the dotted line indicating expected values under a null model. The p-values for the observed relationships are displayed at the top of each graph. (TIF) [file pone.0338441.s010.tif]
